# CryoEM structures of two spliceosomal complexes: starter and dessert at the spliceosome feast

**DOI:** 10.1016/j.sbi.2015.12.005

**Published:** 2016-02

**Authors:** Thi Hoang Duong Nguyen, Wojciech P Galej, Sebastian M Fica, Pei-Chun Lin, Andrew J Newman, Kiyoshi Nagai

**Affiliations:** MRC Laboratory of Molecular Biology, Francis Crick Avenue, Cambridge CB2 0QH UK

## Abstract

The spliceosome is formed on pre-mRNA substrates from five small nuclear ribonucleoprotein particles (U1, U2, U4/U6 and U5 snRNPs), and numerous non-snRNP factors. *Saccharomyces cerevisiae* U4/U6.U5 tri-snRNP comprises U5 snRNA, U4/U6 snRNA duplex and approximately 30 proteins and represents a substantial part of the spliceosome before activation. *Schizosaccharomyces pombe* U2.U6.U5 spliceosomal complex is a post-catalytic intron lariat spliceosome containing U2 and U5 snRNPs, NTC (nineteen complex), NTC-related proteins (NTR), U6 snRNA, and an RNA intron lariat. Two recent papers describe near-complete atomic structures of these complexes based on cryoEM single-particle analysis. The U4/U6.U5 tri-snRNP structure provides crucial insight into the activation mechanism of the spliceosome. The U2.U6.U5 complex reveals the striking architecture of NTC and NTR and important features of the group II intron-like catalytic RNA core remaining after spliced mRNA is released. These two structures greatly advance our understanding of the mechanism of pre-mRNA splicing.

## Highlights

• Recent advances in cryoEM are revolutionizing our understanding of how molecular machines function.
• The structure of *Saccharomyces cerevisiae* U4/U6.U5 tri-snRNP has been revealed.
• The structure of *Schizosaccharomyces pombe* U2.U6.U5 spliceosomal complex has been revealed.
• These structures greatly advanced our understanding of the mechanism of pre-mRNA splicing.

**Current Opinion in Structural Biology** 2016, **36**:48–57

This review comes from a themed issue on **Nucleic acids and their protein complexes**

Edited by **David MJ Lilley** and **Anna Marie Pyle**

For a complete overview see the Issue and the Editorial

Available online 21st January 2016

**http://dx.doi.org/10.1016/j.sbi.2015.12.005**

0959-440/© 2016 The Authors. Published by Elsevier Ltd. This is an open access article under the CC BY license (http://creativecommons.org/licenses/by/4.0/).

## Introduction

**Figure 1:**
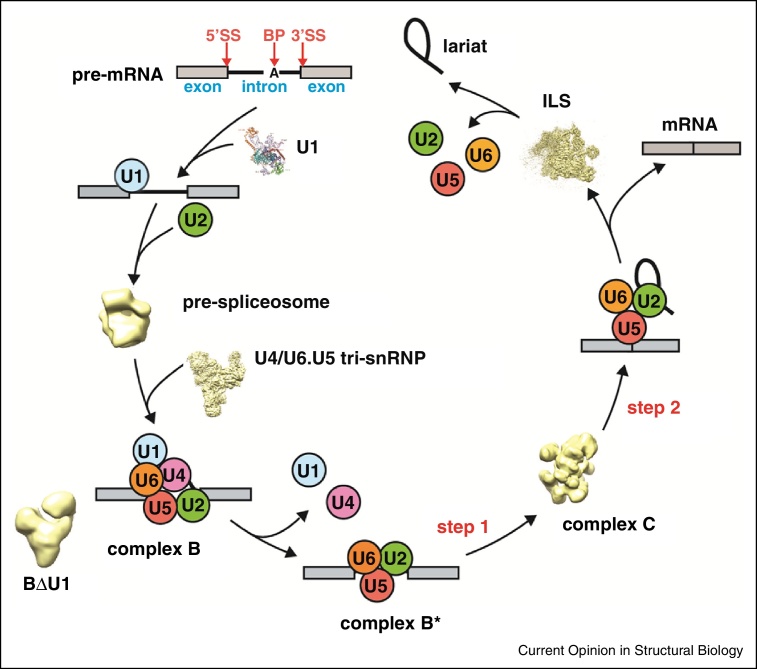
Step-wise spliceosome assembly from its U-snRNP components. The U1 snRNP crystal structure [16, 17•] and available EM maps of some of the complexes are shown: pre-spliceosome (EMDB entry EMD-1325), U4/U6.U5 tri-snRNP [23^••^], BΔU1 [27], complex C [70] and intron lariat spliceosome (ILS) [24^••^].

Much of our current knowledge of the molecular mechanism of pre-mRNA splicing is based on three decades of intensive research using extracts made from the budding yeast *Saccharomyces cerevisiae* or from HeLa cell nuclei [1, 2]. These extracts contain U1 and U2 snRNPs and U4/U6.U5 tri-snRNPs as major spliceosomal components and provide robust systems for monitoring spliceosome assembly and splicing reactions when synthetic pre-mRNA substrates are added [2, 3, 4]. Biochemical studies of these two systems, combined with genetic approaches, have established a universal mechanism of nuclear pre-mRNA splicing (Figure 1). Initially U1 and U2 snRNPs recognise the 5′ splice site and the branch point in pre-mRNA and recruit the pre-assembled U4/U6.U5 tri-snRNP to form the fully assembled but catalytically inactive complex B. Major structural and compositional changes then produce the catalytically active complex B*. These changes include dissociation of U1 snRNP from the 5′ splice site, unwinding of the extensively base-paired U4/U6 snRNA duplex by Brr2 helicase, leading to the loss of U4 snRNA together with its associated proteins, the recruitment of large protein complexes known as NTC (nineteen complex) and NTR (nineteen related complex) [5] and formation of a new base-pairing interaction between U2 and U6 snRNAs that creates an active centre similar to that of group II self-splicing introns [6, 7••, 8••, 9]. These remodelling events position the 2′OH group of the branch point adenosine to attack the phosphodiester bond at the 5′ splice site, producing exon1 and lariat intron-exon2 splicing intermediates. The spliceosome then undergoes further remodelling to become complex C* in which the exons are aligned on the conserved loop 1 of U5 snRNA for the second trans-esterification reaction [10, 11]. The spliced mRNA product is then released and the residual intron lariat spliceosome (ILS) is disassembled, recycling the snRNPs for subsequent rounds of splicing and allowing degradation of the excised intron lariat [4, 12, 13•]. Spliceosomal remodelling events are regulated by several ATP-dependent RNA helicases, whose activities define further conformational states of the spliceosome [14, 15].

The crystal structure of U1 snRNP has been determined [16, 17•] and its mechanism of 5′ splice site selection is now well understood. However, the highly dynamic nature and low abundance of the splicing machinery have prevented crystallisation of other snRNPs or intact spliceosomes. The pioneering EM work of the Stark/Lührmann, Moore/Jurica/Grigorieff and Ohi/Gould/Walz groups has revealed the overall shape of the spliceosome at different assembly steps (Figure 1; reviewed in [18]), and some protein and RNA components have been located within these structures by various labelling techniques [19, 20, 21]. Taking advantage of crucial advances in cryoEM single particle analysis [22] two recent papers report the structure of two spliceosomal complexes, the *Saccharomyces cerevisiae* U4/U6.U5 tri-snRNP [23^••^] and a *Schizosaccharomyces pombe* spliceosomal complex [24^••^], known as endogenous U2·U5·U6 snRNA complex [13^•^] or U5.U2/U6 spliceosome complex [25]. These discoveries have advanced our structural knowledge of the spliceosome enormously.

## Structure of the *S. cerevisiae* U4/U6.U5 tri-snRNP

**Figure 2:**
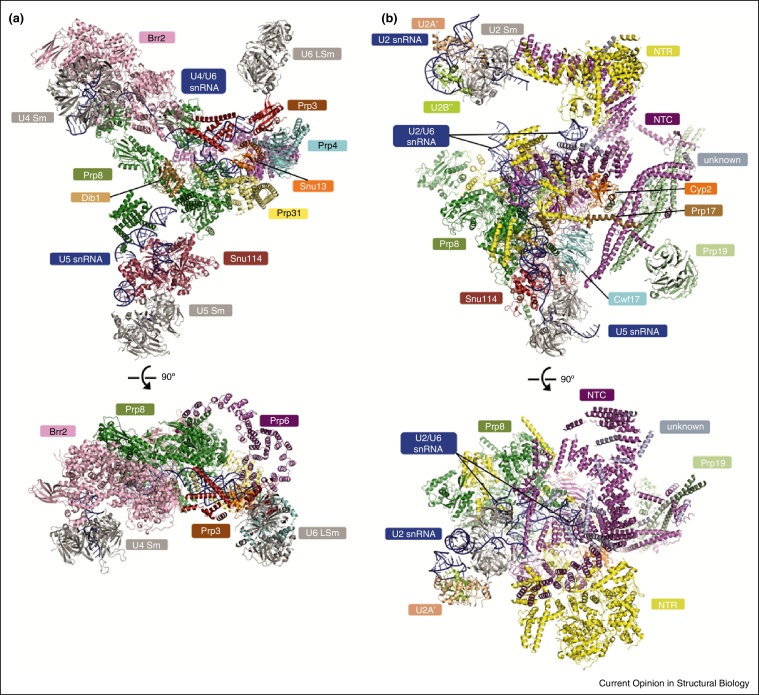
Structure overview of **(a)** the *S. cerevisiae* U4/U6.U5 tri-snRNP and **(b)** the *S. pombe* U2.U6.U5 ILS complex. In both structures, the RNA components are coloured in blue; Prp8, Snu114 and Sm/LSm proteins are coloured in green, red and grey, respectively. The two structures are shown in such a way that the U5 Sm proteins have a similar orientation. (a) The tri-snRNP structure features 29 fitted proteins with U4/U6 and U5 snRNAs. (b) The U2.U6.U5 ILS structure features 37 fitted proteins and U2/U6 and U5 snRNAs. NTC components are all coloured in magenta except for Prp19 being highlighted in light green. NTR components are all coloured in yellow.

At about 1.5 MDa the U4/U6.U5 tri-snRNP is the largest pre-assembled spliceosomal complex [19, 26] and represents a substantial part of the spliceosome before catalytic activation (complex B) [21, 27, 28]. It comprises more than 30 proteins, U5 snRNA and extensively base-paired U4 and U6 snRNAs [29, 30]. A cryoEM map of native affinity-purified yeast U4/U6.U5 tri-snRNP was obtained to an overall resolution of 5.9 Å [23^••^]. The map was of sufficient resolution to fit the crystal structures or homology models of 29 proteins as well as double-stranded snRNA regions, leaving unassigned only a small fraction of the density (Figure 2a). The U5 snRNP components Prp8, Snu114 and Brr2 form a stable complex [31] and play key roles in the activation of the spliceosome and formation of the catalytic centre [32]. The Brr2 helicase contains two helicase cassettes each comprising two RecA, winged helix (WH), Ratchet, helix-loop-helix (HLH), fibronectin3-like (FN3) domains [33, 34, 35, 36, 37]; however only the N-terminal cassette is catalytically active. Brr2 unwinds the U4/U6 snRNA duplex [33], allowing U6 snRNA to form an RNA structure highly similar to the active site of group II self-splicing introns, with the binding sites for two catalytic divalent metal ions [7••, 8••]. Snu114 is a GTPase homologous to eukaryotic elongation factor-2 (EF2) and prokaryotic elongation factor EF-G [38, 39]. It has been suggested that Brr2 is activated when Snu114 is bound to GTP [40] or when GTP is hydrolysed [41].

In U4/U6.U5 tri-snRNP, Prp8, Snu114 and the U5 snRNP core domain occupy the lower part of the triangular assembly, and Brr2 and the U4/U6 di-snRNP occupy the upper part (Figure 2a). Prp8, the largest and most conserved protein in the spliceosome [32], is located at the centre of the assembly and acts as a hub for RNA–protein and protein–protein interactions. The crystal structure of Prp8 residues 885–2413 [42], in complex with the assembly factor Aar2 [43], revealed the ‘large domain’, consisting of Reverse Transcriptase-like (RT), thumb/X, linker and Type II restriction endonuclease-like domains [42]. The large domain is connected to the RNaseH-like and the Jab1/MPN domains with disordered linkers of approximately 10 and 70 residues, respectively. Aar2 restrains these three domains into a stable assembly. In U4/U6.U5 tri-snRNP Aar2 is not present [37] and both the RNaseH and Jab1/MPN domains are released from the large domain. These two domains could change positions and orientations further in different spliceosomal complexes and interact with different protein and RNA components [42] (Figure 2a). In U4/U6.U5 tri-snRNP, the RNaseH-like domain rotates with respect to the large domain while the Jab1/MPN domain, which can form a stable complex with Brr2 [37, 44], moves more than 120 Å and interacts with the Endonuclease-like domain. The tri-snRNP structure revealed that the N-terminal domain of Prp8 is predominantly α-helical and stably associates with Snu114 [45, 46] and U5 snRNA stem-loop 1.

**Figure 3:**
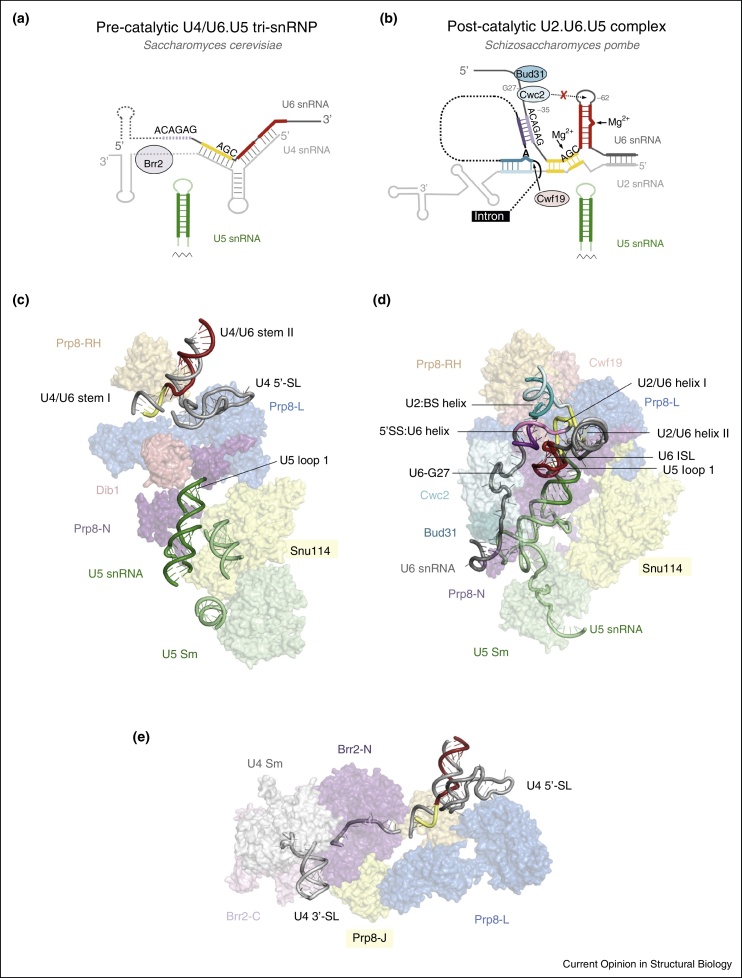
Comparison of the proteins and RNAs at the core of the U4/U6.U5 tri-snRNP and U2.U6.U5 spliceosomal complex. **(a)** Schematic representation of the RNA secondary structure in U4/U6.U5 tri-snRNP; **(b)** Schematic representation of the RNA secondary structure and selected proteins contacting the RNA in U2.U6.U5 spliceosomal complex. Cwc2 strongly cross-links with U6 snRNA ISL in *S. cerevisiae* and human complex C [66] but in *S. pombe* U2.U6.U5 spliceosomal complex Cwc2(*Cwf2*) is not in contact with U6 ISL [24^••^]; **(c)** Core structure of the U4/U6.U5 tri-snRNP; **(d)** Core structure of the U2.U6.U5 spliceosomal complex; **(e)** Brr2 loading onto U4 snRNA in the U4/U6.U5 tri-snRNP. Prp8-J, Jab1/MPN domain of Prp8; Prp8-RH, RNaseH-like domain of Prp8; Prp8-L, large domain of Prp8; Prp8-N, N-terminus of Prp8; BS, branch site; 5′SS, 5′-splice site; ISL, internal stem-loop; SL, stem-loop. In (c) and (d) the large domain of Prp8 is in the same orientation.

In the upper part of tri-snRNP U4 and U6 snRNAs are extensively base-paired, with U4-U6 stems I and II coaxially stacked (Figure 3): Snu13 binds to the k-turn motif of U4 snRNA 5′ stem-loop inducing further assembly of Prp31 and the Prp3-Prp4 dimer [47, 48, 49]. The WD40 domain of Prp4 [50] interacts with the ferredoxin-like domain of Prp3, which in turn binds the single stranded region of U6 snRNA [51^•^] and contacts the Lsm core domain bound to the 3′ end of U6 snRNA. Finally, Prp6 forms a striking α-solenoid structure connecting Snu13 and Prp4 with the RNaseH-like domain of Prp8 (Figure 2a). Comparison with the low-resolution structure of complex B shows that U2 snRNP interacts with this region of U4/U6.U5 tri-snRNP either directly or indirectly [21, 27, 28]. Brr2 forms a stable complex with the Jab1/MPN domain of Prp8 [37, 44], which is connected to its RNaseH-like domain through a 70-residue flexible linker peptide. *In vitro* experiments suggested that Brr2 loads onto the single stranded region of U4 snRNA between the 3′ stem-loop and stem I of the U4/U6 snRNA duplex and translocates along U4 snRNA [52, 53]. Mozaffari-Jovin *et al*. [53] proposed that the Prp8 RNaseH domain binds to the forked single-stranded region preceding U4/U6 stem I and prevents the loading of Brr2 onto U4 snRNA. In the U4/U6.U5 tri-snRNP this single stranded region of U4 snRNA is already loaded into the active site of the N-terminal helicase cassette of Brr2 ready for unwinding by translocation along U4 snRNA (Figure 2, Figure 3). Upon addition of ATP, U4/U6.U5 tri-snRNP is disassembled as a consequence of the U4/U6 snRNA duplex unwinding by Brr2 [23^••^].

Prp8 crosslinks to 4-thiouridine introduced at key positions in U6 snRNA, the invariant exon-binding loop 1 of U5 snRNA and at all three sites of chemistry in the pre-mRNA (5**′**-SS, branch point and 3**′**-SS) showing that Prp8 interacts intimately with the catalytic RNA core of the spliceosome [54]. The crosslinks of the pre-mRNA branch point (BP + 2) in the catalytically active spliceosome map to the region between Prp8 residues 1585 and 1598 (C.M. Norman and A.J.N., unpublished result), which is located on the surface of the RT Thumb/X and linker domains, the most positively charged and conserved surface of Prp8. This surface is part of a cavity that could accommodate the group II intron-like catalytic RNA core and hence we proposed that this region forms the active site cavity of the spliceosome [42]. In the U4/U6.U5 tri-snRNP structure, the conserved loop 1 of U5 snRNA, which aligns the exons in the second catalytic step [10, 11], is inserted into the active site cavity and points into the most electropositive and conserved surface of Prp8 in the Thumb/X and linker region (Figure 3c). This suggests that part of the active site is pre-assembled in the tri-snRNP and that Prp8 provides a platform for docking the other RNA components at the catalytic core.

## Structure of the *S. pombe* U2.U6.U5 spliceosomal complex

Although fission yeast, *Schizosaccharomyces pombe*, has been used extensively to study various aspects of eukaryotic cell functions, its use for the study of pre-mRNA splicing has been limited as it has not been possible to prepare active splicing extract. In *S. pombe* cell extracts an endogenous complex containing U2, U6 and U5 snRNAs (hereafter referred to as ‘U2.U6.U5 spliceosomal complex’) is found as an abundant component. Ohi *et al.* [25] purified this complex using TAP-tagged Cdc5, an NTC component, and presented a 29 Å resolution EM structure. The Moore/Query group purified U2.U6.U5 spliceosomal complex using a split TAP-tag approach and carried out comprehensive characterisation of its protein and RNA components [13^•^]. They estimated the molecular mass of this complex to be approximately 2.5 MDa. On the basis of RNAseq analysis they concluded that this complex is an ILS. Interestingly the Brr2 helicase, crucial for U4/U6 snRNA unwinding during activation, is under-represented in this complex even in low salt and completely missing in high salt. Brr2 helicase is also implicated in unwinding U2/U6 snRNA duplex during spliceosomal disassembly [40], perhaps explaining how ILS accumulates in *S. pombe* extract.

**Figure 4:**
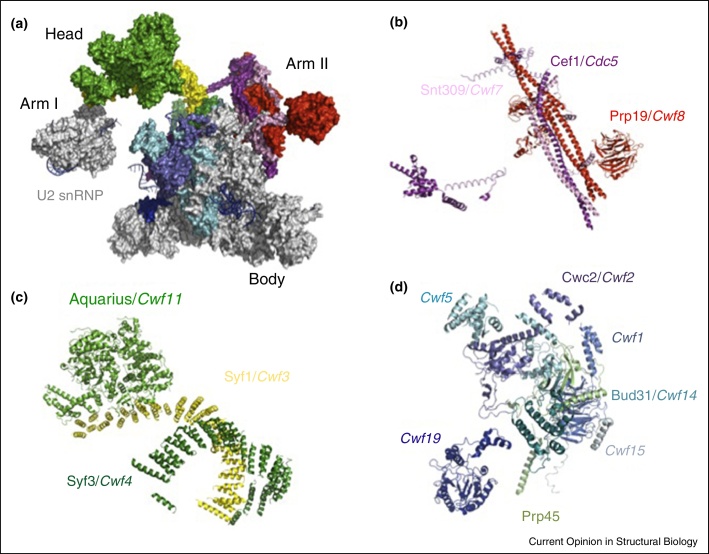
Structure of NTC and NTR. **(a)** The distribution of NTC and NTR components within the U2.U6.U5 spliceosome, comprising Body, Head, Arm I and Arm II domains. The Arm II, Head and Body regions are highlighted in red, green and blue, respectively. **(b)** The structures of NTC components, Prp19, Cef1 and Snt309 in the Arm II domain. Prp19 (red) forms a tetramer via its coiled-coil helices and U-box, and further interacts with Cef1 (purple) and Snt309 (pink). **(c)** Aquarius (green) and two superhelical proteins Syf1 (yellow) and Syf3 (dark green) form the Head domain protruding from the main body of the U2.U6.U5 spliceosomal complex. **(d)** The structures of NTC and NTR components in the Body region. Two proteins, Cwc2 (*Cwf2*) and *Cwf19*, shown in light and dark blue, respectively, are found close to the catalytic centre. Cwc2 was shown to interact with U6 snRNA ISL and induce an active conformation of the spliceosome's catalytic RNA elements [66]. In the U2.U6.U5 spliceosomal complex Cwc2 no longer interacts with U6 snRNA ISL. *Cwf19* shows homology to *S. cerevisiae* Drn1 which interacts with the debranching enzyme Dbr1 [65^•^].

Yan *et al*. [24^••^] recently reported a 3.6 Å resolution structure of the U2.U6.U5 spliceosomal complex purified using a protocol based on Ohi *et al*. [25] and modeled the snRNAs, the lariat intron and 37 proteins corresponding to a combined molecular mass of approximately 1.3 MDa (Figure 2b) [55^••^]. Distinct protein names have been used for *S. pombe* splicing factors; for clarity, the more familiar human/*S. cerevisiae* nomenclature will be used below alongside italicized *S. pombe* nomenclature (see [13•, 56]). The structure was divided into Body, Head and Arms I and II (Figure 2, Figure 4). Arm I comprises a subdomain of U2 snRNP comprising U2B″/U2A′ and the core domain consisting of seven Sm proteins from which U2 snRNA extends into the active site cavity. The most notable feature of Arm II is a helix bundle composed of three NTC components — Prp19(*Cwf8*), Snt309(*Cwf7*) and Cef1(*Cdc5*) — providing a first glimpse of the remarkable architecture of NTC. Prp19(*Cwf8*) [57], known as a key factor for NTC assembly [56, 58, 59, 60, 61], forms a tetramer via its U-box and coiled-coil domains [60] although only one of the WD40 domains is ordered [62] (Figure 4). The long α-helices of Snt309(*Cwf7*) and the C-terminal region of Cef1(*Cdc5*) interact with the coiled coil region of Prp19(*Cwf8*) whereas the N-terminal region of Cef1(*Cdc5*) reaches the RT domain of Prp8(*Spp42*). In the Head domain long arched α-helical solenoids of Syf1(*Cwf3*) and Syf3(*Cwf4*) form a cross creating a basket-like structure while Aquarius(*Cwf11*) — comprising armadillo and helicase domains [63^•^] — binds to one arm of Syf1(*Cwf3*). Aquarius(*Cwf11*) is integrated into the spliceosome as part of intron binding complex (IBC) which crosslinks with components of U2 snRNP [63^•^]. The Aquarius(*Cwf11*) ATPase is activated by RNA but its precise role in splicing is unknown. In the U2.U6.U5 spliceosomal complex Aquarius(*Cwf11*) is located between U2 snRNP and Syf1(*Cwf3*) and their interaction is mediated or strengthened by Syf1(*Cwf3*) and Isy1 [63^•^].

In the Body domain Prp8(*Spp42*), Snu114(*Cwf10*), and the U5 core domain are arranged essentially in the same way as in the *S. cerevisiae* U4/U6.U5 tri-snRNP (Figure 2, Figure 3) but loop 1 of U5 snRNA has moved slightly deeper into the active site cavity. As in U4/U6.U5 tri-snRNP, stem-loop 1 of U5 snRNA points into the most positively charged and conserved surface of the Thumb/X and linker domains of Prp8(*Spp42*) where the BP + 2 nucleotide crosslinks with an amino acid on this surface (between residues 1585 and 1598 of *S. cerevisiae* Prp8) in the active spliceosome (C.M. Norman and A.J.N., unpublished result) [42]. After the U4/U6.U5 tri-snRNP joins complex A, U1 snRNP is displaced from the 5′ splice site and the ACAGA sequence in U6 snRNA pairs with the 5′-splice site. During spliceosomal activation Brr2 unwinds the U4/U6 snRNA duplex and U4 snRNA together with Snu13, Prp31 and Prp3/Prp4 dissociate from the spliceosome, causing U6 snRNA to be dramatically restructured (Figure 3).

**Figure 5:**
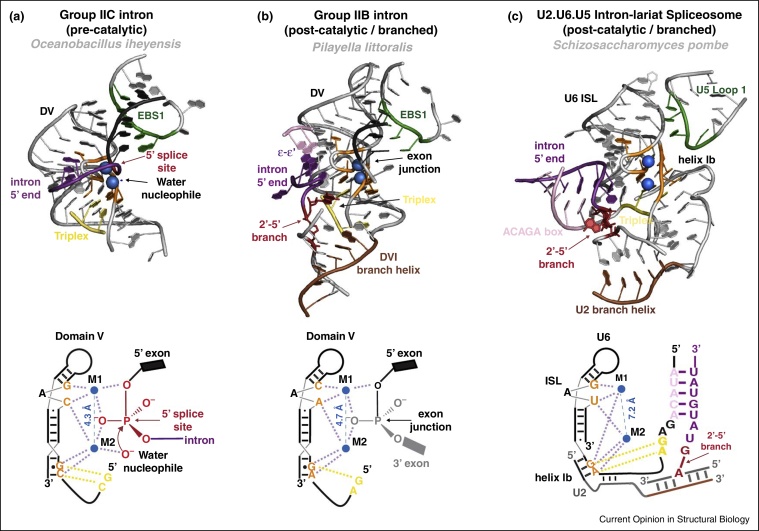
Comparison of the RNA core of the U2.U6.U5 spliceosomal complex to the catalytic core of group II self-splicing introns. **(a)** Structure of the catalytic domain V of a group IIC intron trapped in the pre-catalytic state in the presence of Ca^2+^ (PDB 4FAQ) [71]. The 5′ splice site scissile phosphate is aligned with the two metals bound at the core in a catalytic configuration, as shown in the lower diagram. Note that the nucleophile is a water molecule, rather than the branch adenosine 2′OH in group IIB and spliceosomal introns. **(b)** Structure of the RNA core of a group IIB intron trapped in a post-catalytic, branched configuration, with the ligated exons bound at the core (PDB 4R0D) [72]. Note that the interactions between domain V and the catalytic metals are conserved between the group IIC and group IIB structures (compare to a). **(c)** Structure of the RNA core of the U2.U6.U5 spliceosomal complex in a post-catalytic configuration (PDB 3JB9) [24^••^], probably following release of the mRNA. Note that the interactions between U6 ligands and the two Mg^2+^ ions at the core are slightly re-organized compared to the group II intron structures. Lower panels show schematic representations of the structures, including key interactions between the catalytic metals and the reactive phosphates in the group II structures. Residues that position the catalytic metals are shown in orange and the catalytic metals are coloured light blue, while residues that form the third strand of the triplex and their interactions are shown in yellow.

Whereas in the U4/U6.U5 tri-snRNP U6 snRNA is close to the RNase H-like domain of Prp8, in the U2.U6.U5 complex U6 has been repositioned into the active site cavity formed by the Prp8(*Spp42*) large and N-terminal domains (Figure 3). Here U6 snRNA forms extensive base pairs with U2 snRNA to produce a group II intron-like catalytic centre (Figure 5) [6, 7••, 9]. Consistent with previous genetic and biochemical studies [6, 8••], U2 and U6 form a triplex that brings the U6 ISL and the AGC triad into close proximity and allows U6 to bind two Mg^2+^ ions for catalysis (Figure 5c). The triplex configuration in the U2.U6.U5 complex is similar to that observed for domain V in pre-catalytic and post-catalytic structures of the group II intron (Figure 5a–c). The 5′ splice site/ACAGA helix is perpendicular to the U2 branch helix — a configuration that mimics the position of the 5′ splice site ɛ–ɛ′ helix with respect to the DVI branch helix of group II introns (Figure 5b,c). However, the 2′–5′ branch linkage is 20 Å removed from the two Mg^2+^ ions bound by the U6 triplex (Figure 5c). A similar displacement of the 5′ splice site is seen in the post-catalytic group II structure, underscoring the post-catalytic configuration of the U2.U6.U5 complex (Figure 5b,c). By contrast, in the pre-catalytic group II structure, the 5′ splice site is positioned in proximity to the two catalytic Mg^2+^ ions and the 5′ exon is aligned on the EBS1 loop (Figure 5a,b). Although in the U2.U6.U5 complex U5 loop 1 is positioned similarly to EBS1 in the group II structures, the exon junction was not observed in the U2.U6.U5 cryo-EM density, probably because the mRNA has already been released [13^•^]. As a result, the U6 metal ligands reorganize and the two Mg^2+^ ions are placed more than 7 Å apart [55^••^] — a significant displacement from the 4 Å spacing preferred for phosphoryl transfer catalysis [64] (compare Figure 5c and a).

In the U2.U6.U5 spliceosomal complex the C-terminal domain of *Cwf19* (Cwf19L2 in human and no apparent orthologue found in *S. cerevisiae*) fills the space between the large and RNaseH-like domains of Prp8(*Spp42*), inserting an extension of its Zn-finger domain into the active centre [24^••^]. The C-terminal domain of *S. cerevisiae* protein Drn1 shows significant similarity to the C-terminal domain of *Cwf19*. Its N-terminal domain is known to interact with the debranching enzyme Dbr1 and Syf1(*Cwf3*) [65^•^]. It is tempting to suggest that Drn1 binds to Prp8(*Spp42*) in the post-splicing complex and recruits Dbr1 to the lariat intron after spliced mRNA and step 2 factors dissociate from the active centre.

U6 snRNA extends across U5 snRNA to the N-terminal domain of Prp8(*Spp42*). Its 3′ end forms a duplex with the 5′ end of U2 snRNA. Bud31(*Cwf14*) bound to the N-terminal domain of Prp8(*Spp42*) anchors the 5′ end of U6 snRNA while Cwc2(*Cwf2*) is bound to the adjacent single stranded region of U6 snRNA. Cwc2(*Cwf2*) promotes formation of the group II intron-like structure of the catalytic centre [8••, 66, 67]. In *S. cerevisiae* B^act^ and C complexes Cwc2 cross-links with U6 snRNA internal stem-loop (ISL) and G39 (G27 in *S. pombe* U6 snRNA) upstream of the ACAGAGA sequence (Figure 3); in human the Cwc2 orthologue RBM22 crosslinks with the equivalent region of U6 snRNA [66]. G39 is in contact with Cwc2(*Cwf2*) in the U2.U6.U5 spliceosomal complex structure but Cwc2(*Cwf2*) is far away from U6 ISL (Figure 3b and d). In the U2.U6.U5 spliceosomal complex U6 ISL is exposed and could make contact with Cwc2(*Cwf2*) if it is rotated. None of the step 2 factors (Slu7, Prp18, Prp22) are found in this structure showing that these proteins have already dissociated from the complex [4, 68]. Therefore the structure shows a number of characteristics of the post-splicing ILS complex and it is unlikely to be complex C or C*. Burke *et al*. [69] showed by NMR and SAXS that U2/U6 does not spontaneously form a group II intron-like RNA structure in isolation even in the presence of Mg^2+^. The fact that in U2.U6.U5 spliceosomal complex the U2/U6 snRNA pair forms a metal-binding triple helical structure (Figure 5c) implies that once this structure has formed the active site cavity is sufficient to maintain its integrity even in the absence of step 2 factors and disruption of the interaction between Cwc2(*Cwf2*) and U6 ISL.

## Conclusions

The U4/U6.U5 tri-snRNP revealed the structure of the spliceosome before activation and provided important insight into the activation mechanism and the role of Prp8 in formation of the active centre. The U2.U6.U5 spliceosomal complex structure most probably represents the ILS complex after spliced mRNA release but still retains some important characteristics of the active spliceosome. It also provided a first glimpse of NTC and NTR and how they interact with the spliceosomal snRNPs. Much remains to be understood about the different conformational states of this dynamic RNP machine and how these states are regulated by trans-acting ATPases. The recent cryo-EM structures have paved the way for detailed structural analysis of the spliceosome and the field can look forward to many new exciting structures.

## Conflict of interest

Nothing to declare.

## References and recommended reading

Papers of particular interest, published within the period of review, have been highlighted as:

• of special interest
•• of outstanding interest
